# Accounting for indirect protection in the benefit–risk ratio estimation of rotavirus vaccination in children under the age of 5 years, France, 2018

**DOI:** 10.2807/1560-7917.ES.2020.25.33.1900538

**Published:** 2020-08-20

**Authors:** Sylvie Escolano, Judith E Mueller, Pascale Tubert-Bitter

**Affiliations:** 1 Université Paris-Saclay, UVSQ, Univ. Paris-Sud, Inserm, High-Dimensional Biostatistics for Drug Safety and Genomics, CESP, Villejuif, France; 2 EHESP French School of Public Health, Paris, France; 3 Institut Pasteur, Paris, France

**Keywords:** Rotavirus vaccine, benefit-risk ratio, indirect protection, simulation analysis, rotavirus gastroenteritis, intussusception

## Abstract

**Background:**

Rotavirus is a major cause of severe gastroenteritis in children worldwide. The disease burden has been substantially reduced in countries where rotavirus vaccines are used. Given the risk of vaccine-induced intussusception, the benefit–risk balance of rotavirus vaccination has been assessed in several countries, however mostly without considering indirect protection effects.

**Aim:**

We performed a benefit–risk analysis of rotavirus vaccination accounting for indirect protection in France among the 2018 population of children under the age of 5 years.

**Methods:**

To incorporate indirect protection effects in the benefit formula, we adopted a pseudo-vaccine approach involving mathematical approximation and used a simulation design to provide uncertainty intervals. We derived background incidence distributions from quasi-exhaustive health claim data. We examined different coverage levels and assumptions regarding the waning effects and intussusception case fatality rate.

**Results:**

With the current vaccination coverage of < 10%, the indirect effectiveness was estimated at 6.4% (+/− 0.4). For each hospitalisation for intussusception, 277.0 (95% uncertainty interval: (165.0–462.1)) hospitalisations for rotavirus gastroenteritis were prevented. Should 90% of infants be vaccinated, indirect effectiveness would reach 57.9% (+/− 3.7) and the benefit–risk ratio would be 192.4 (95% uncertainty interval: 116.4–321.3). At a coverage level of 50%, indirect protection accounted for 27% of the prevented rotavirus gastroenteritis cases. The balance remained in favour of the vaccine even in a scenario with a high assumption for intussusception case fatality.

**Conclusions:**

These findings contribute to a better assessment of the rotavirus vaccine benefit–risk balance.

## Introduction

Rotavirus infections are responsible for severe diarrhoea and vomiting in children, including substantial case fatality if appropriate care cannot be provided. The World Health Organization (WHO) estimated that during the pre-vaccination era, more than 2 million children worldwide were hospitalised each year for rotavirus gastroenteritis (RVGE) [1]. Oral live attenuated rotavirus vaccines have been introduced in more than 90 countries to date and substantial reductions in disease burden have been observed [2]. For high-income countries with high vaccine coverage (VC) such as some European countries and the United States (US), a large reduction in the number of hospitalisations for acute gastroenteritis is considered attributable to the vaccine [3,4]. Two vaccines are currently marketed globally: Rotarix (a monovalent, two-dose schedule vaccine) and Rotateq (a pentavalent, three-dose schedule vaccine), all doses being administrated before the age of 8 months. Post-marketing surveillance and analyses based either on epidemiological studies or on pharmacovigilance data have shown an increased but limited risk of intussusception, especially during the first week after administration of the first dose, but also possibly during the second and third weeks and after the second dose [5-10].

In the context of increasing coverage, the transmission of the virus to susceptible persons becomes a rarer event. In addition, because vaccinated children transmit the virus to contacts to a lesser extent, the vaccine has the potential to indirectly protect unvaccinated persons. Based on both effects, a vaccination programme with high VC can provide indirect or herd protection, and eventually herd immunity, a situation where no new cases occur. The importance of the indirect protection effect depends on various factors that are specific to the virus (transmissibility, asymptomatic forms of infection), the vaccine (level of serum antibody response and capacity to induce mucosal immunity) and the population (contact patterns, conditions of hygiene, VC). It is therefore difficult to estimate indirect protection effects in a vaccination programme. Analysis of population surveillance data in terms of incidence changes in out-of-target age groups and unvaccinated individuals following vaccine introduction may suggest the presence of indirection protection and allow a rough estimate of its strength. However, for precise estimates, clinical trials with specific designs are required [11].

Several studies in countries possessing long-term surveillance data and a range of high VC have reported substantial indirect protection effects, although the effect estimates varied between studies, countries and age groups [12-15]. Such variation makes it challenging to include vaccine-induced indirect protection effects in predictive modelling.

The public health impact of rotavirus vaccination has been assessed in several middle- and high-income countries by estimating benefit–risk (BR) ratios. These evaluations can be conducted for different vaccine scenarios, they can be used to quantify the current impact of vaccination or to predict the impact of immunisation programme changes. Therefore, they apply to the current local setting but also to hypothetical ones, typically with varying levels of VC. To provide uncertainty intervals (UI), BR studies may also involve model-generated simulations [16-20]. However, to assess the overall population impact of a vaccination, it is necessary to estimate the benefit including indirect protection effects. So far this has not been attempted in rotavirus BR studies, except for a recent analysis conducted in the Netherlands [21].

Rotarix and RotaTeq have been marketed in France since 2006 and 2007, respectively, but in the absence of a recommendation by health authorities or reimbursement, VC was estimated to be less than 10% according to a survey conducted between 2008 and 2013 [22]. In 2013, the French national technical committee on vaccination recommended rotavirus vaccination, conditional on a future cost-effectiveness evaluation. In 2015, however, two cases of intussusception with delayed care and fatal outcome were observed and the recommendation was withdrawn [23]. We previously estimated the BR ratio for rotavirus vaccination in the 2015 population in France at a median value of 214 for hospitalisations and 273 for deaths [20]. That analysis did not take the effects of indirect protection into account. In the present paper, we propose a new evaluation of this BR ratio that now includes it. Highly variable indirect effect estimates exist in high-income countries with medium-to-high VC and none is available for low-coverage settings, yet indirect effectiveness is expected to decrease with lower coverage levels. To circumvent these difficulties and to use realistic values in all scenarios, we used an approximated mathematical equation relating indirect effectiveness and VC that was proposed by Bauch et al. [24]. It involves an epidemiological metric: the basic reproduction number of an infection. In our approach, we considered this number as an additional parameter for which estimates for rotavirus infection are available in several high-income countries.

Overall, we aimed at estimating the BR ratio for the French population in various VC scenarios. We extended our model for BR ratio estimation to incorporate indirect protection in the algorithm and obtained corresponding predictions of indirect effectiveness. Finally, we also aimed at exploring the impact of assuming a higher case fatality of intussusception.

## Methods

### General study design and data sources

We developed an extended version of the model presented in Lamrani et al. [20]. The general purpose was to quantify the benefits of rotavirus vaccines (defined as the yearly number of prevented hospitalisations or deaths for RVGE in children under the age of 5 years), their risks (the yearly number of induced hospitalisations or deaths for intussusception in children under the age of 1 year), and then to calculate the ratio of these two estimates. This was done with a simulation study and applied to the French population in 2018. For parameterisation, we aimed at including French data wherever available or approached the situation in France with transposable data from other settings. Key parameters were (i) epidemiological and demographical features (i.e. number of children under 1 year and under 5 years of age living in France and VC), (ii) relative risk (RR) of intussusception in the 3 weeks following administration of a vaccine dose and (iii) vaccine efficacy, including direct and indirect effects.

Although the main difference with our previous work was that we took the effects of indirect protection into account [20], other enhancements were made. Firstly, to fit the underlying age distribution of RVGE and of intussusception, we used exhaustive data from the French national health care system, rather than a sample. Together with patient age, this database included all hospitalisations occurring in children under the age of 5 years from 2009 to 2015 in France that were coded as K56.1 (for intussusceptions) and A08.0 (for RVGE) according to the ICD-10 classification. Secondly, we introduced a multiplicative correction factor for the incidence of RVGE and intussusception, taking into consideration the fact that the national estimates comprised a (small) number of vaccinated cases, whereas we wanted to estimate the background incidences (Supplementary Methods SM1 and SM2). Without these correction factors, the incidence of intussusception would have been slightly overestimated and the incidence of RVGE would have been slightly underestimated because vaccination induces some intussusception cases and prevents some RVGE cases. Thirdly, we modified the assumption about the long-term duration of protection after immunisation by exploring several waning scenarios after the 3rd year of life, as main or sensitivity analyses. Finally, we updated demographical data using 2018 values for French populations of children under 1 year of age and under 5 years of age (711,904 and 3,726,091, respectively [25]).

Three levels of VC were explored: 10%, the current approximate coverage which is considered as the base scenario in this work, 50%, a coverage level reached in many countries where the vaccine is recommended and realistic for rotavirus vaccine introduction without specific communication or reinforcement, and 90%, the observed coverage of recommended infant vaccines in France. Based on French pharmacy sales data, we assumed that 70% of administered doses were Rotarix and 30% Rotateq in the base scenario [22].

Using Monte Carlo simulations, we sampled parameters independently according to their distribution (Supplementary Tables S1 and S2) and generated simultaneous estimates of the number of cases avoided and induced, and the according benefit-risk ratios. Simulations were iterated 20,000 times. With this approach, point estimates are given as the 50% percentiles (i.e. median values) and UI are given as the 2.5% and 97.5% percentiles of the distributions resulting from the simulations. The model was written in SAS language and we used SAS 9.4 version to perform the simulations. 

### Modelling

#### Vaccine benefit assessment accounting for indirect protection

At the population level, the benefit of an immunisation programme is due to direct and indirect protection. In this work, the indirect protection was accounted for by introducing a ‘pseudo-vaccine’, which we assumed covered the entire child population with equal benefit and without any adverse event. Among unvaccinated children, this indirect effectiveness *E^I^* applied homogeneously regardless of age. Among vaccinated children, it applied alone before receipt of the first dose of vaccine, while after the first dose, it applied in combination with direct efficacy *E^D^*. Thus, vaccinated children benefitted from total effectiveness *E^T^* [11], where

$$\;\;\;\;\;\;\;\;\;\;\;\;\;\;\;\;\;\;\;\;\;\;\;\;1-E^{T}=\left(1-E^{D}\right)\left(1-E^{I}\right)\;\;\;\;\;\;\;\;\;\;\;\;\;\;\;\;\;\;\;\;\;\;\;\;\;\;\;\;\;\;\;\;\;\;\;\;\;\;\;\;\;\;\;\;\;\;\;\;\;(1)\;\;\;$$

**Figure 1:**
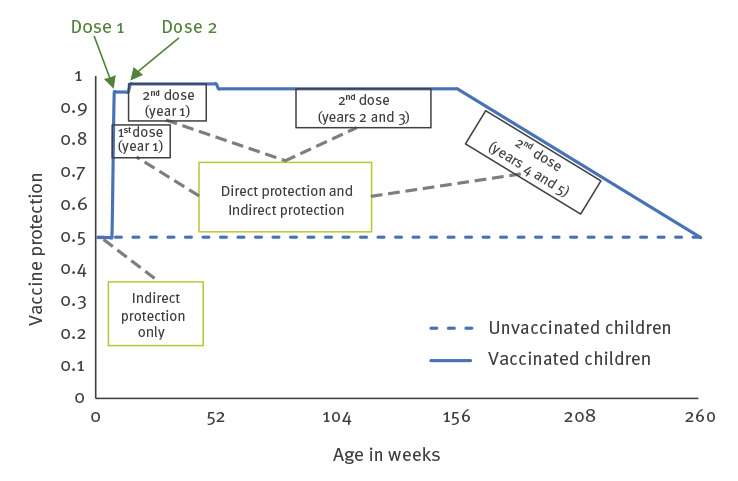
Illustration of rotavirus vaccine protection against gastroenteritis in children

The parameter for direct protection corresponded to the vaccine efficacy estimated in clinical trials, with vaccine protection decreasing during the first 3 years following immunisation [26]. Consequently, we assumed that children were protected by the vaccine as soon as the first dose was administered and that this protection remained constant until another dose was administered or until the end of the first year of life. During the 2nd and 3rd years of life, children continued to benefit from direct protection, albeit at a lower level because of waning of antibodies. During the 4th and 5th years of life, we assumed in the base scenario that vaccine efficacy linearly waned to zero (Figure 1).

This illustrative example corresponds to a fictitious population with 90% vaccine coverage, indirect protection of 50% and linear waning of antibodies, where all vaccinated children receive two doses of vaccines (in the 7th and 14th weeks of age), have direct effectiveness of 90% after dose 1, of 95% after dose 2 (during the 1st year) and 92% (during the 2nd and the 3rd year). Then, according to Formula (1), the total effectiveness for vaccinated children is 50% before week 7 and after week 260, 95% between weeks 7 and 14, 97.5% between weeks 14 and 52 and 96% between weeks 52 and 156.

#### Built-in indirect effectiveness

The estimates of the indirect protection effect available in the literature were observed in populations with a coverage of ca 50% or higher. In Germany, a 48% indirect effectiveness was estimated given a coverage of 47.6% (mean values over the observed period 2007–2017 [12]). In a meta-analysis, the VC ranged from 54.1% (in 2007–2008, US) to 93% (in 2013–2014, United Kingdom (UK)) [14]. In France, the VC is substantially lower and no pre-vaccine data are available to estimate the indirect protection effect. Therefore, for the three levels of VC, indirect protection levels were derived from Formula (2) which proposes an approximation of indirect effectiveness *E^I^* from the VC, the direct efficacy *E^D^* and the basic reproduction number *R*_0_ (average number of secondary infections per primary case in a susceptible population) for universal vaccination against a paediatric infectious disease [24]:

$${\;\;\;\;\;\;\;\;\;\;\;\;\;\;\;\;\;\;\;\;\;\;\;\;\;\;\;\;\;\;\;\;\;\;\;E}^{I}\approx \frac{R_{0}\times VC\times E^{D}}{R_{0}-1}\;\;\;\;\;\;\;\;\;\;\;\;\;\;\;\;\;\;\;\;\;\;\;\;\;\;\;\;\;\;\;\;\;\;\;\;(2)\;$$

According to Formula (2), *E^I^* decreases with *R*_0_ (for *R*_0_ > 1) with minimum VC × *E^D^*. Rotavirus is highly infectious with *R*_0_ estimates ranging from 11 to 54 in children younger than 5 years in high-income countries [27,28]. Although in Formula (2), the value of *R*_0_ has a modest impact on *E^I^*, as opposed to VC and *E^D^*, we chose an overdispersed discrete distribution of *R*_0_ to cover this range of estimates (Supplementary Table S1, last line). As for *E^D^* in Formula (2), an average direct efficacy over both vaccines, doses and ages *E^D^* was used (Supplementary Method SM1). We used this value *E^I^* for unvaccinated children and in Formula (1) for vaccinated children.

#### Benefit–risk ratio calculation

Details on formulas for benefit and risk calculations are given in the Supplementary Methods. The benefit is the annual number of prevented hospitalisations for RVGE and depends on the background number of infants hospitalised at age *w* (*w* = 1 to 261 weeks), on the proportion of the population newly vaccinated by dose *d* of either vaccine at age w, on *E^T^* for either vaccine at dose *d* in week *t* of vaccination and on *E^I^*. The risk is the annual number of vaccine-induced hospitalisations for intussusception and depends on the background number of infants experiencing the adverse event at age *w* (*w* = 1 to 52 weeks), the proportion vaccinated by dose *d* of either vaccine at age *w* and the RR of intussusception in week *t* after dose *d* of vaccination with either vaccine. Finally, the BR ratio is simply obtained by dividing the benefit by the risk. Similar calculations apply for deaths.

### Sensitivity analyses

Concerning the duration of protection, we considered two opposite scenarios: (i) accelerated waning, meaning that direct efficacy was not maintained beyond the 3rd year of life, so that the protection of a vaccinated child fell back to the indirect protection level by the age of 4 years and (ii) absence of waning, so that direct efficacy at 2 years of age was maintained until the age of 5 years.

For purposes of comparison, we performed a set of simulations without any indirect protection. We performed another set of simulations based on an assumption that only Rotarix or only Rotateq were available in the market.

Finally, we considered the conservative assumption where the case fatality rate for intussusceptions would reach the highest value among the countries covered by the review paper on childhood intussusception (i.e. 0.7%, observed in Spain) [29]. For this scenario, we made the most conservative choice for the persistence of vaccine efficacy, assuming accelerated waning.

### Ethical statement

Because this was a simulation study, ethical approval was not needed.

## Results

### Background incidences

The annual background number of hospitalised RVGE in France was estimated at a median of 11,400 (95% UI: 8,770–14,500) and the annual background number of hospitalised intussusceptions at 192 (95% UI: 167–218). Age distributions are displayed in Supplementary Figures S2 and S3. Thus, the corresponding incidences were 3.1 per 1,000 for RGVE in children younger than 5 years and 2.8 per 10,000 for intussusceptions in children younger than 1 year.

### Built-in indirect effectiveness estimates

With the three chosen VC rates (10%, 50% and 90%), the indirect effectiveness *E^I^* as calculated from Formula (2) was estimated at a mean of 6.4% (standard deviation (SD): +/− 0.4), 32.2% (SD: +/− 2.0) and 57.9% (SD: +/− 3.7), respectively (Table 1). The distributions obtained after 20,000 simulations are displayed in Supplementary Figure S1.

**Table 1:** Estimated indirect effectiveness and annual benefits and risks of rotavirus vaccine, under various scenarios of vaccine coverage and efficacy waning, France, 2018 (n = 20,000 simulations)

Vaccine coverage	Waning scenario	Indirect effectiveness, mean (SD)	Benefit: number of prevented rotavirus gastroenteritis cases		Risk: number of induced intussusceptions		Benefit–risk ratio	
			Median	95% uncertainty interval ^a^	Median	95% uncertainty interval ^a^	Median	95% uncertainty interval ^a^
10%	Linear	6.4% (0.4)	1,686 *2.3*	1,274 – 2,173 *2.0* – *2.6*	6.1 *0.006*	3.9 – 9.3 *0.001* – *0.02*	277.0 *371.5*	165.0 – 462.1 *123.0 – 1,697*
	Accelerated	5.5% (0.3)	1,546 *2.1*	1,169 – 1,192 *1.9* – *2.4*			254.6 *345.3*	152.1 – 428.6 *113.2 – 1,600*
	Absence	7.3% (0.5)	1,796 *2.5*	1,355 – 2,337 2.1 – 2.8			295.4 *398.6*	177.0 – 496.7 *134.0* – *1,799*
50%	Linear	32.2% (2.0)	7,120 *9.8*	5,416 – 9,170 *8.7 – 10.9*	30.3 *0.03*	19.3 – 46.3 *0.007 – 0.09*	234.4 *317.0*	141.3 – 391.0 *106.5 – 1,425*
	Accelerated	27.7% (1.6)	6,677 *9.1*	5,088 – 8,599 *8.2 – 10.2*			219.7 *296.8*	133.3 – 364.1 *98.3 – 1,361*
	Absence	36.7% (2.4)	7,478 *10.3*	5,662 – 9,634 *9.1* – *11.5*			247.1 *331.1*	147.8 *–* 411.4 *110.4 – 1,484*
90%	Linear	57.9% (3.7)	10,500 *14.5*	8,050 – 13,420 *13.0 – 15.9*	54.6 *0.05*	35.0 – 83.8 *0.01 – 0.2*	192.4 *257.8*	116.4 – 321.3 *87.0 – 1,209*
	Accelerated	49.9% (3.0)	10,100 *13.9*	7,730 – 12,940 *12.5 – 15.4*			184.9 *247.3*	112.1 – 305.5 *83.7 – 1,132*
	Absence	65.9% (4.3)	10,780 *14.8*	8,256 *–* 13, *13.4 – 16.3*			197.9 *266.4*	120.3 *–* 327.8 *88.6 – 1,231*

SD: standard deviation.

^a^ 2.5%–97.5% percentiles.

All simulations assumed a mixture of 70% Rotarix and 30% Rotateq vaccines. Benefits, risks and benefit–risk ratios are given for hospitalisation (standard font) and for death (italic font).

### Benefit–risk ratio estimates

In the base scenario where coverage was 10% with an assumption of linear waning of antibodies, we estimated a median BR ratio of 277.0 (95% UI: 166.7–44.5) for hospitalisations and 371.5 (95% UI: 123.0–1,697) for deaths (Table 1). The BR ratio decreased with VC: for 50%, we estimated a BR ratio of 234.5 (95% UI: 141.3–391.0) for hospitalisations and 317.0 (95% UI: 106.5–1,425) for deaths and for a coverage of 90%, a BR ratio of 192.4 (116.4–321.3) for hospitalisations and 257.8 (95% UI: 87.0–1,209) for deaths (Table 1). While the estimated indirect effectiveness increased with VC, predicted BR ratios in our model decreased with larger VC.

### Estimated impact of indirect protection

**Figure 2:**
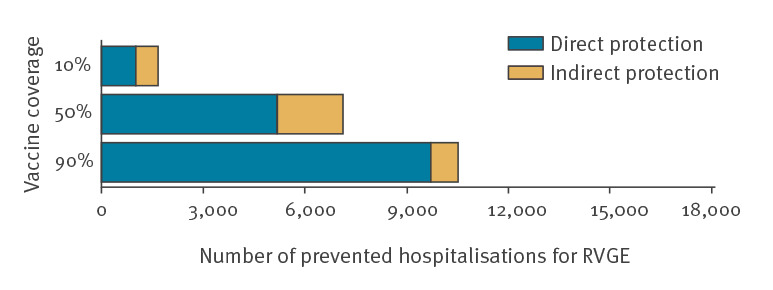
Number of hospitalisations prevented by direct and indirect protection, obtained under several scenarios of rotavirus vaccine coverage, France, 2018 (n = 20,000 simulations)

Without including any indirect protection effect, we estimated a BR ratio of 164.4 (95% UI: 98.5–272.6) for hospitalisations and 221.5 (95% UI: 73.4–1,003) for deaths (Table 2). The proportion of prevented hospitalisations for RVGE thanks to indirect protection decreased with higher VC levels, from 40.2% (for 10% coverage) to 26.8% (for 50% coverage) and 7.6% (for 90% coverage) (Figure 2).

**Table 2:** Estimated annual benefits and benefit-risk ratios of rotavirus vaccine, under various scenarios of vaccine coverage and efficacy waning, scenario without indirect protection, France 2018 (n = 20,000 simulations)

Vaccine coverage	Waning scenario	Benefit: Number of prevented rotavirus gastroenteritis episodes		Benefit–risk ratio^a^	
		Median	95% uncertainty interval^b^	Median	95% uncertainty interval^b^
10%	Linear	998.4 *1.4*	756.1 – 1,280 *1.2 – 1.5*	164.4 *221.5*	98.5 *–* 272.6 *73.4 – 1,003*
	Accelerated	956.9 *1.3*	728.1 – 1,230 *1.2 – 1.5*	158.0 *214.9*	95.4 *–* 261.7 *70.6 – 967.2*
	Absence	1,018 *1.4*	772.9 *–* 1,310 1.2 *–* 1.6	167.8 *223.7*	101.1 – 281.9 *73.7 – 1,031*
50%	Linear	4,990 *6.9*	3,800 – 6,420 *6.1 – 7.7*	*^a^*	*^a^*
	Accelerated	4,780 *6.6*	3,630 – 6,140 *5.8 – 7.3*		
	Absence	5,100 *7.0*	3,890 – 6,560 *6.2 – 7.8*		
90%	Linear	8,970 *12.4*	6,830 – 11,540 *10.9 – 13.8*	*^a^*	*^a^*
	Accelerated	8,610 *11.9*	6,550 – 11,070 *10.5 – 13.2*		
	Absence	9,160 *12.6*	6,960 *–* 11,820 *11.1 – 14.1*		

^a^ Benefit–risk ratio does not depend on vaccine coverage in the event that there is no indirect protection.

^b^ 2.5%–97.5% percentiles.

All simulations always assumed a mixture of 70% Rotarix and 30% Rotateq vaccines. Benefits, risks and benefit–risk ratios are given for hospitalisation (standard font) and for death (italic font).

RVGE: rotavirus gastroenteritis.

### Sensitivity analyses

The estimates for indirect effectiveness were slightly lower when assuming accelerated waning. For 10%, 50% and 90% VC, we obtained 5.5%, 27.7% and 49.9%, respectively (Table 1 and Supplementary Figure S1). Likewise, BR ratios were slightly lower; for 10% coverage for example, they were 254.6 (95% UI: 152.1–428.6) for hospitalisations and 345.3 (95% UI: 113.2–1,600) for deaths (Table 1). In case of absence of waning, the corresponding estimates were slightly higher: 7.3%, 36.7% and 65.9% for indirect effectiveness in the three CV scenarios, and 295.4 (95% UI: 177.0–496.7) and 398.6 (95% UI: 134.0–1,799) for the BR ratios for hospitalisations and deaths, respectively, with 10% coverage.

The whole set of simulations was also run for scenarios where only Rotarix or only Rotateq were available and the corresponding results are displayed in Supplementary Tables S4a and S4b for simulations with indirect protection and in Supplementary Tables S5a and S5b for simulations without indirect protection effect. We observed very marginal changes compared with the results of the scenario with a mixture of Rotarix and Rotateq vaccines.

**Figure 3:**
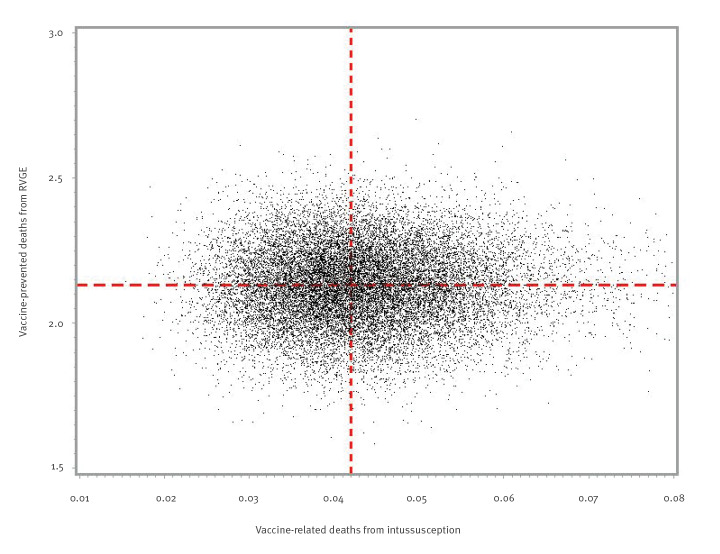
Number of vaccine-prevented deaths from rotavirus gastroenteritis in children under 5 years of age (benefit) vs vaccine-related deaths from intussusception in infants under 1 year of age (risk), France, 2018 (n = 20,000 simulations)

In addition, assuming a 0.7% case fatality rate, accelerated waning and 10% coverage, the annual number of prevented deaths from RVGE was 2.1 (95% UI: 1.9–2.4) with an annual number of vaccine-induced deaths from intussusception of 0.042 (95% UI: 0.027–0.065) (Figure 3). The BR ratio for deaths in this scenario was 50.1 (95% UI: 32.3–79.7, declining to 30.9 (95% UI: 19.9–48.9) if no indirect protection effect was included.

RVGE: rotavirus gastroenteritis.

Assumptions: a high estimate of case fatality (0.7%) for intussusceptions, 10% coverage and accelerated waning effect.

## Discussion

The goal of this study was to provide an accurate and comprehensive BR assessment of rotavirus vaccination in France, by comparing the number of RVGE hospitalisations (or deaths) prevented by the vaccination with the number of hospitalisations (or deaths) induced by intussusception as an effect of the vaccination; we also included an indirect protection effect as a specific model component that varied across a range of VC scenarios. For VC of 10% (current), 50% and 90% (potential coverage rates), we found that the BR ratio ranged from 192 to 277 for hospitalisations and from 258 to 371 for deaths. Our results indicate that it decreases with VC and that the contribution of indirect protection effects to the benefit also decreases with VC.. At a coverage level of 50%, indirect protection accounted for about a quarter of the prevented rotavirus gastroenteritis cases. Sensitivity analyses showed that the alternative assumptions on waning only marginally impacted the results. Furthermore, a substantial BR ratio persisted under the unfavourable assumption of higher case fatality associated with intussusception, with a lower BR ratio uncertainty interval limit at 32.3. Another specificity of this work is that background incidences of RVGE and intussusceptions were calculated by using exhaustive data from the French national healthcare system and corrective factors, and that it mimics the French context (market distribution between Rotarix and Rotateq).

Because no indirect effect had been included in our previous work [20], the benefits and the BR ratios resulting from the present analyses are greater than those already published, even for more conservative assumptions about the waning of antibodies. The BR ratio without indirect immunity obtained in the present work could be considered as the lower limit of the BR, in case indirect protection would be negligible. The only available BR study accounting for indirect protection found a BR ratio estimate of 685 hospitalisations with a hypothetical 86% VC in the Netherlands [21]. This ratio is larger than what we observed in our study (198 with no waning and 90% coverage), even though the authors applied an indirect effectiveness of 30% maximum, which is lower than the 66% estimated in our approach for this coverage. The gap between these results is mainly driven by the choice in the risk component: Bruijning et al. used one excess case of intussusception per 50,000 vaccinated children, which is by far more optimistic than ours. Of note, they also ran simulations using one excess intussusception case per 20,000 vaccinated children and obtained then a much lower BR value of 274. Compared with estimates from other countries (see Table 3 in reference [19] for example), our BR ratio estimates without indirect protection effects were lower than the range of published values for hospitalisations (from 282 in Mexico to 1,265 in Brazil) and fall within the range of published values for deaths (from 71 in the US to 395 in Latin America).

In the present study, we approximated indirect effectiveness by using a formula including coverage, direct effect and *R*_0_. Although the dynamics of rotavirus infection are not fully understood and *R*_0_ estimates vary widely, there is evidence that the basic reproduction number for rotavirus is high. Another difficulty is that the approximation proposed by Bauch et al. assumes lifelong vaccine-derived and natural immunity, which may not be met for rotavirus. At the same time, as mentioned by the authors, this approximation only partially accounts for indirect protection or herd immunity [24]. Despite these challenges, the indirect effectiveness estimates that we produced and used fall within the range of estimates derived from real-life surveillance data in populations with ca 50% VC (e.g. in the US) or 90% VC (e.g. in Belgium, Australia or Great Britain) [12-15].

In mathematical modelling, indirect protection effects are usually taken into account by using dynamic transmission models, which produce indirect effects depending on hypotheses about age-specific mixing patterns and risk of transmission [27]. Such a model was developed for evaluating the cost effectiveness of Rotateq vaccination in France [30], assuming 75% VC, but the indirect effectiveness was not explicitly quantified in that work. In addition, results from some mathematical modelling studies on rotavirus predict a limited indirect protection effect that contrasts with the large reductions in incidence in unvaccinated age groups observed in countries with high coverage levels ([31], p. 32).

In this work, we had to make several simplifying assumptions. Firstly, the pseudo-vaccine approach supposes that the vaccine has been on the market long enough and the coverage is stable. Secondly, the possible interactions of children younger than 5 years with other age groups, whether with older children (6–10 years) or with adults, were not taken into account. Thirdly, the population of children under the age of 5 years was considered as a whole, which means that we did not introduce age-specific indirect effects. However, a clear relationship between age and the amount of indirect protection has not yet been established. Comparing the results of studies performed in three high-income countries among children under 5 years of age, estimates from the US were highest for the youngest and decreased with age, estimates in Great Britain were constant across age groups, while estimates in Australia were high in the middle-aged group (36–47 months) and low in other age groups [14]. Fourthly, estimation of RVGE and intussusception incidences and age distributions were not based on epidemiological surveillance but on national drug claims and hospital discharge data, by identifying cases through specific ICD10 codes. Fifthly, for some of the input parameters, we could not find values resulting from French studies. Wherever possible, we tried to input results from studies performed in Europe or at least in high-income countries. Finally, the approximation proposed by Bauch et al. was obtained with a pseudo-dynamic model by including the basic reproduction number *R*_0_, a transmission feature of the rotavirus [24]. We acknowledge that this static approach oversimplifies the likely complex pattern of the disease. In sensitivity analyses, the BR ratio estimation was overall robust and not dependent on assumptions of efficacy persistence. Additional knowledge about the effectiveness of rotavirus vaccines would help refine the proposed modelling framework.

BR ratios without indirect protection effects may be the most relevant for vaccine decision by families and doctors for individual children. However, as soon as the goal of protecting vulnerable persons or eliminating rotavirus disease is established, the BR ratio including indirect protection becomes more relevant, even for individual decision-making. From the perspective of national decision makers, BR estimates including indirect protection are the most relevant, and our results suggest that the benefits of recommending vaccination against rotavirus outweigh the risks. However, some additional considerations may be required before implementing nationwide recommendations or obligations. Firstly, for currently recommended or mandatory infant vaccines in France, the risk of a severe and possibly fatal side effect can be estimated at 0.0003% for the paediatric hexavalent vaccine (anaphylactic shock) and 0.0022% for the measles-mumps-rubella vaccine (adding up risk estimates for anaphylactic shock, encephalitis and thrombocytopenic purpura) [32-36]. This is substantially lower than our estimate of ca 0.0086% for the rotavirus vaccine. As French people are keenly aware of vaccine safety [37], they may not agree with the claim that ‘rotavirus vaccine is safe’. Secondly, the tendency of parents to attribute a more negative value to vaccine-induced than disease-induced deaths, also known as the omission bias, has been described for several recommended vaccinations [38,39]. Similarly, averting the side effects of vaccines was found to dominate judgments in vaccine decision-making among adults in the UK [40]. Such an individual preference could limit acceptance of the rotavirus vaccine despite official recommendations. As safety concerns interact with the perception of disease risk [41], BR analyses give structure to the implicit reasoning of individuals and society at large. In any case, national decisions about vaccine recommendations need to be based not only on scientific data but also on political and societal priorities.

## Conclusion

The BR ratio estimates for rotavirus vaccination are substantially impacted by taking into account indirect protection effects. We have simulated indirect protection effects from rotavirus vaccination with simple techniques, yielding estimates that are roughly comparable to those obtained with data from surveillance studies. Given the major uncertainty about the exact level of indirect protection effects, these modelling techniques have helped to mitigate knowledge gaps about the full impact of vaccination at the population level for different coverage scenarios. We used a simulation framework to incorporate the uncertainty of the model parameters into the estimation and carefully considered relevant sources of uncertainty. Addressing these issues is an important step towards an unbiased assessment of the BR ratios of vaccination. This adds stronger evidence on which decision-making and communication in vaccination programmes can be based. 

## Authors’ correction

After publication, the authors informed us on 24 August that a computational error had occurred in the calculation of indirect benefit. An Expression of Concern was published on the same day.

The affected results have been recalculated and the article was replaced with the corrected version on 1 October 2020.

The corrections affect the numbers for indirect benefit in the following parts of the manuscript:

• Abstract

• Manuscript text

• Table 1

• Figure 2

• Figure 3

• Supplement

We thank the authors for informing us swiftly about the error and for the efforts in correcting it.

## Supplementary Data

1057000 Supplement
