# Expression of concern for Euro Surveill. 2020;25(33)

**DOI:** 10.2807/1560-7917.ES.2020.25.34.200827ec

**Published:** 2020-08-27

**Authors:** Eurosurveillance editorial team

**Affiliations:** 1 European Centre for Disease Prevention and Control (ECDC), Stockholm, Sweden

Regarding the article "Accounting for indirect protection in the benefit–risk ratio estimation of rotavirus vaccination in children under the age of 5 years, France, 2018" by Escolano et al, published on 20 August 2020, *Eurosurveillance* has been informed of a computational error that has occurred in the calculation of indirect benefit. The authors are in the process of recalculating the affected results. 

While we await these results and the extent of the necessary corrections, the editors decided to publish an expression of concern for this article.

