# Resolved: Expression of concern for Euro Surveill. 2020;25(32)

**DOI:** 10.2807/1560-7917.ES.2020.25.39.201001en

**Published:** 2020-10-01

**Authors:** Eurosurveillance editorial team

**Affiliations:** 1 European Centre for Disease Prevention and Control (ECDC), Stockholm, Sweden

A computational error occurred in the calculation of indirect benefit in the article "Accounting for indirect protection in the benefit–risk ratio estimation of rotavirus vaccination in children under the age of 5 years, France, 2018" by Escolano et al, published on 20 August 2020.

The authors informed *Eurosurveillance* of the error on 24 August and an Expression of Concern was published on the same day.

The corrections were screened by the two peer original reviewers as well as an Associate editor with the relevant expertise. Based on their views and confirmation that the corrections do not have a major bearing on the overall results and the main conclusions of the article, the *Eurosurveillance* editors have concluded that a retraction of the article is not necessary.

The article was replaced with a corrected version on 1 October 2020, in which the affected results have been recalculated. An Authors’ correction is published in the same issue. 

We thank the authors for informing us swiftly about the error and for the efforts in correcting it, and the reviewers for taking the time to ensure the quality of the changes. 

