# Authors’ correction for Euro Surveill. 2020;25(32)

**DOI:** 10.2807/1560-7917.ES.2020.25.39.201001c

**Published:** 2020-10-01

**Authors:** Eurosurveillance editorial team

**Affiliations:** 1 European Centre for Disease Prevention and Control (ECDC), Stockholm, Sweden

A computational error occurred in the calculation of indirect benefit in the article "Accounting for indirect protection in the benefit–risk ratio estimation of rotavirus vaccination in children under the age of 5 years, France, 2018" by Escolano et al, published on 20 August 2020.

The authors informed *Eurosurveillance* of the error on 24 August and an Expression of Concern was published on the same day.

The affected results have been recalculated and the article was replaced with the corrected version on 1 October 2020. 

The corrections affect the numbers for indirect benefit in the following parts of the manuscript:

- Abstract
- Manuscript text
- Table 1
- Figure 2
- Figure 3
- Supplement

We thank the authors for informing us swiftly about the error and for the efforts in correcting it. 

An accompanying Editorial note outlines our actions following the Expression of concern that led to the decision to correct the article. 

