# Supplementary material for: Accounting for indirect protection in the benefit–risk ratio estimation of rotavirus vaccination in children under the age of 5 years, France, 2018
Source: Euro Surveill. 2020 Aug 20;25(33):1900538. doi: 10.2807/1560-7917.ES.2020.25.33.1900538 (PMC7441603; doi:10.2807/1560-7917.ES.2020.25.33.1900538)
Supplement: Supplement [file 19-00538_ESCOLANO_Supplement.pdf]

SUPPLEMENTARY MATERIAL

This supplementary material is hosted by *Eurosurveillance* as supporting information alongside the article '*Accounting for indirect protection in the benefit-risk ratio estimation of rotavirus vaccination in children under 5 years of age, in France*' on behalf of the authors, who remain responsible for the accuracy and appropriateness of the content. The same standards for ethics, copyright, attributions and permissions as for the article apply. Supplements are not edited by *Eurosurveillance* and the journal is not responsible for the maintenance of any links or email addresses provided therein.

Supplement Methods

SM1 Multiplicative correction factor for rotavirus gastroenteritis background incidence and benefit calculations

This factor is:

$$corrRVGE = \frac{1}{1 - f \times E^{O}}$$

where:

- f = 0.095, the proportion of vaccinated children at the time of the survey (1),

- E^{O} is the overall effectiveness (2): $E^{O} = 1 - (1 - E^{I})(1 - f \times \overline{E^{D}})$ where E^{I} stands for the indirect effectiveness as calculated from f, R_0 and $\overline{E^{D}}$ according to formula (2) in the manuscript, and $\overline{E^{D}}$ is an average value for direct efficacy, computed as explained below:

$$\overline{E^{D}} = 0.7\,\overline{E^{rx}} + 0.3\,\overline{E^{rq}} \quad (2.1)$$

$$\overline{E^{rx}} = 0.04\,\overline{E_1^{rx}} + 0.96\,\overline{E_2^{rx}} \quad (2.2a)$$

$$\overline{E^{rq}} = 0.04\,\overline{E_1^{rq}} + 0.96 \times 0.04\,\overline{E_2^{rq}} + 0.96 \times 0.96\,\overline{E_3^{rq}} \quad (2.2b)$$

$$\overline{E_d^{rx}} = \tfrac{1}{5}E_{d,1}^{rx} + \tfrac{2}{5}E_{d,2\&3}^{rx} + \tfrac{2}{5}E_{d,4\&5}^{rx}\text{ , for } d\text{=1 to 2} \quad (2.3a)$$

$$\overline{E_d^{rq}} = \tfrac{1}{5}E_{d,1}^{rq} + \tfrac{2}{5}E_{d,2\&3}^{rq} + \tfrac{2}{5}E_{d,4\&5}^{rq}\text{ , for } d\text{=1 to 3} \quad (2.3b)$$

where

- $\overline{E_d^{rx}}$ (resp. $\overline{E_d^{rq}}$) stands for the average direct efficacy of the dth dose of Rotarix (resp RotaTeq)

- $E_{d,1}^{rx}$, $E_{d,2\&3}^{rx}$ and $E_{d,4\&5}^{rx}$ stands for the direct efficacy of the dth dose of Rotarix during the 1st year of life, during the 2nd and 3rd years of life and during the 4th and 5th year of life. Similar notations with "*rq*" instead of "*rx*" superscript were used for RotaTeq.

Note that

- in the main analyses (linear waning), $E^{rx}_{d,4\&5} = \frac{E^{rx}_{d,2\&3}}{2}$ and $E^{rq}_{d,4\&5} = \frac{E^{rq}_{d,2\&3}}{2}$, whereas in the sensitivity analyses (accelerated waning) $E^{rx}_{d,4\&5} = 0$ and $E^{rq}_{d,4\&5} = 0$.

- Eq (2.1) states that in the French population, the vaccine can be either Rotarix (70%) or RotaTeq (30%).

- Eq (2.2a) and (2.2b) state that the vaccination schedule is not necessarily complete (we assumed that only 96% of children who received a given dose will receive the following one, following Sabbe's observations (3)).

- Eq (2.3a) and (2.3b) state that efficacy of the dth dose of Rotarix (resp. RotaTeq) depends on the age of the children. Three periods have been considered: the first period corresponds to the first year of life, whatever the actual age of the child at the first dose.

- E^I is an averaged parameter; in particular, it does not depend on age.

For each simulation, $corrRVGE$ is derived from *f*, which is fixed, and from all direct efficacies, that is, $E^{rx}_{d,\tau}$ and $E^{rq}_{d,\tau}$, *d* = 1 to 3 and τ = 1, 2&3, 4&5 which are sampled.

This correction factor is then introduced in the benefit calculation. Let $H(w)$ be the observed number of infants hospitalized for RVGE at age w. $H(w)$ is the product of the sampled annual incidence of RVGE (see Supplement Table S1) and the fixed proportion of cases at age *w* (see Supplement Figure S2). Then $H_0(w) = corrRVGE \times H(w)$ is the background number of children hospitalized for RVGE at age w.

The benefit depends:

- in unvaccinated children: on indirect effectiveness E^I,

- in vaccinated children: on the total effectiveness (dependent on time, vaccine name and dose): $E^{T,rx}_{d,\tau}$ and $E^{T,rq}_{d,\tau}$, *d* = 1 to 3 and τ = 1, 2&3, 4&5, according to formula (1) in the manuscript,

where E^I is computed with formula (2) of the manuscript, from the chosen vaccine coverage VC and from the sampled R_0 and direct efficacies $E^{rx}_{d,\tau}$ and $E^{rq}_{d,\tau}$, *d* = 1 to 3 and τ = 1, 2&3, 4&5 using the same averaging principle as the one described in equations (2.1) to (2.3b).

Overall, for each simulation, the algorithm for calculating all efficacies and effectiveness is:

1. Sample the basic reproduction number and direct efficacies for each vaccine, all doses and every time period.
2. Compute the average direct efficacy from direct efficacies.
3. Compute the indirect effectiveness (from the average direct efficacy and the basic reproduction number
4. Compute total efficacies for each vaccine, all doses and every time period, from corresponding direct efficacies and indirect effectiveness.

Then the benefit (prevented hospitalizations) B_H is calculated as:

$$B_H = \sum_{w=1}^{261} H_0(w) \left\{ \sum_{i=1}^{w} \Big[v_1^{rx}(i) E_1^{T,rx}(w-i+1) \right.$$
$$+ v_2^{rx}(i) \left[E_2^{T,rx}(w-i+1) - E_1^{T,rx}(w-i+1) \right]$$
$$+ v_1^{rq}(i) E_1^{T,rq}(w-i+1)$$
$$+ v_2^{rq}(i) \left[E_2^{T,rq}(w-i+1) - E_1^{T,rq}(w-i+1) \right]$$
$$+ v_3^{rq}(i) \left[E_3^{T,rq}(w-i+1) - E_2^{T,rq}(w-i+1) \right] \Big]$$
$$\left. + \left[1 - \sum_{i=1}^{w} \left(v_1^{rx}(i) + v_1^{rq}(i) \right) \right] E^I \right\}$$

where v_d^{rx}(w) (resp v_d^{rq}(w)) is the proportion of the population vaccinated by dose *d* of Rotarix (resp RotaTeq) at age *w* (see Supplement Table S3 for details).

Finally, B_H is the annual number of prevented hospitalizations for RVGE and $B_D = B_H \times CFR_{RVGE}$ the number of prevented deaths from RVGE (where CFR_{RVGE} is the case fatality rate of RVGE, calculated as the ratio of the number of deaths over the number of hospitalizations from RVGE per year in children under 5).

SM2 Multiplicative correction factor for intussusception background incidence and risk calculations

This factor is the attributable fraction due to vaccine exposure:

$$corrIS = \frac{1}{1 + f(\overline{RR} - 1)}$$

where:

- f =0.095 is the proportion of vaccinated children at the time of the survey (1),

- $\overline{RR}$ is the relative risk, here, the average risk to which children are exposed until the age of 5 years old, computed as explained below:

$$\overline{RR} = 0.7\,\overline{RR^{rx}} + 0.3\,\overline{RR^{rq}} \quad (1.1)$$

$$\overline{RR^{rx}} = 0.04\,\overline{RR_1^{rx}} + 0.96\,\overline{RR_2^{rx}} \quad (1.2a)$$

$$\overline{RR^{rq}} = 0.04\,\overline{RR_1^{rq}} + 0.96 \times 0.04\,\overline{RR_2^{rq}} + 0.96 \times 0.96\,\overline{RR_3^{rq}} \quad (1.2b)$$

$$\overline{RR_1^{rx}} = \frac{1}{261}\left[\sum_{t=1}^{3} RR_1^{rx}(t) + (261 - 3) \times 1\right] \quad (1.3a)$$

$$\overline{RR_2^{rx}} = \frac{1}{261}\left[\sum_{t=1}^{3} RR_1^{rx}(t) + \sum_{t=1}^{3} RR_2^{rx}(t) + (261 - 6) \times 1\right] \quad (1.3b)$$

$$\overline{RR_1^{rq}} = \frac{1}{261}\left[\sum_{t=1}^{3} RR_1^{rq}(t) + (261 - 3) \times 1\right] \quad (1.3c)$$

$$\overline{RR_2^{rq}} = \frac{1}{261}\left[\sum_{t=1}^{3} RR_1^{rq}(t) + \sum_{t=1}^{3} RR_2^{rq}(t) + (261 - 6) \times 1\right] \quad (1.3d)$$

$$\overline{RR_3^{rq}} = \overline{RR_2^{rq}} \quad (1.3e)$$

where

- $\overline{RR_d^{rx}}$ (resp. $\overline{RR_d^{rq}}$) stands for the average relative risk for children having received the *d* first doses of Rotarix (resp RotaTeq)

- $RR_d^{rx}(t)$ (resp. $RR_d^{rq}(t)$) stands for the relative risk for children having received the dth dose of Rotarix (resp RotaTeq) in the tth week.

Note that:

- Eq (1.1) states that in the French population, the vaccine can be either Rotarix (70%) or RotaTeq (30%).

- Eq (1.2a) and (1.2b) state that the vaccination schedule is not necessarily complete (we assumed that only 96% of children who received a given dose will receive the following one).

- Eq (1.3a) and (1.3c) state that the vaccine-induced risk occurs only during the 3 weeks following the first dose, so the risk equals 1 during the $261 - 3$ remaining weeks for those having received one dose.

- Eq (1.3b) and (1.3d) state that the vaccine-induced risk occurs during the 3 weeks following the first dose and during the 3 weeks following the second dose, so the risk equals 1 during the $261 - 6$ remaining weeks for those having received two doses.

- Eq (1.3e) states that the risk is similar for children having received 2 or 3 doses of RotaTeq, because the third dose has no vaccine-induced risk, ie, $RR_3^{rq}(t) = 1$.

For each simulation, $corrIS$ is derived from *f*, which is fixed, and from all vaccine-induced relative risks, that is, $RR_d^{rx}(t)$ and $RR_d^{rq}(t)$, *d* = 1, 2 and *t* = 1 to 3, which are sampled.

This correction factor is then introduced in the risk calculation. Let $A(w)$ be the observed number of infants experiencing the adverse event at age *w*. $A(w)$ is the product of the sampled annual incidence of IS (see Supplement Table S2) and the fixed proportion of cases at age *w* (see Supplement Figure S3). Then $A_0(w) = corrIS \times A(w)$ is the background number of children experiencing the adverse event at age w.

$$R_H = \sum_{w=1}^{52} \left[v_1^{rx}(\mathrm{w}) \sum_{t=1}^{3} A_0(w+t-1)[RR_1^{rx}(t) - 1] + v_2^{rx}(\mathrm{w}) \sum_{t=1}^{3} A_0(w+t-1)[RR_2^{rx}(t) - 1] \right.$$
$$\left. + v_1^{rq}(\mathrm{w}) \sum_{t=1}^{3} A_0(w+t-1)\left[RR_1^{rq}(t) - 1\right] + v_2^{rq}(\mathrm{w}) \sum_{t=1}^{3} A_0(w+t-1)\left[RR_2^{rq}(t) - 1\right] \right]$$

where v_d^{rx}(w) (resp v_d^{rq}(w)) is the proportion of the population vaccinated by dose *d* of Rotarix (resp RotaTeq) at age *w* (see Supplement Table S3 for details).

Finally, R_H is the annual number of vaccine-induced hospitalisations for intussusception, and $R_D = R_H \times CFR_{IS}$ the number of vaccine-induced deaths from intussusception (where CFR_{IS} is the case fatality rate of intussusception).

Supplement Figures

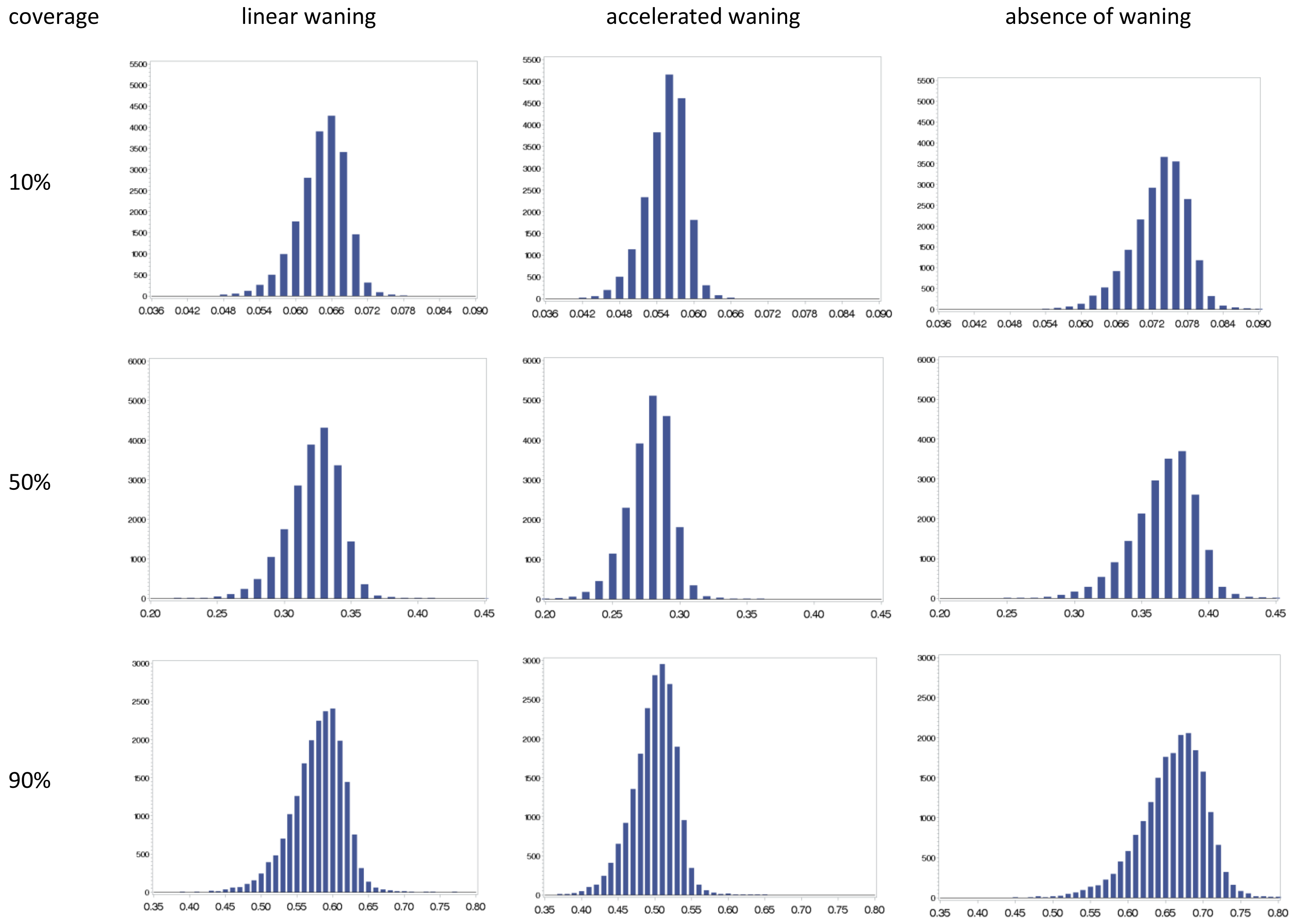

Figure S1: Distribution of indirect effectiveness according to coverage (10%, 50% and 90%) and waning scenario (linear; accelerated, absence)

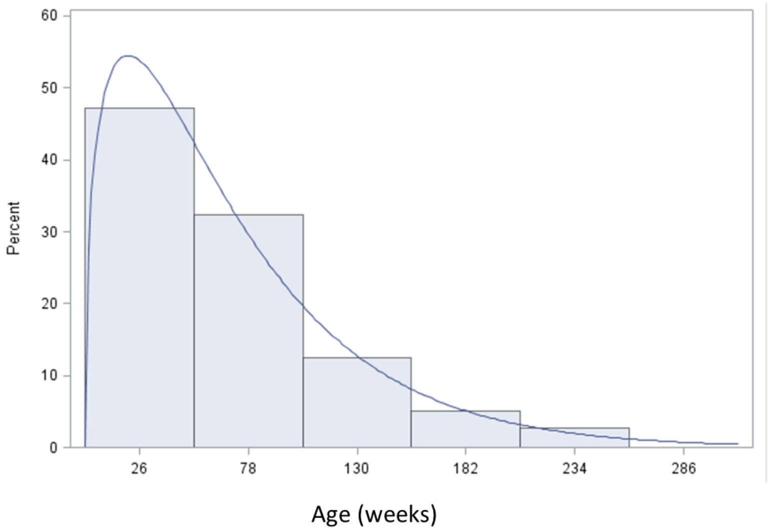

Figure S2: Age distribution of hospitalizations for rotavirus gastroenteritis fitted from the hospital stays coded A08.0 and registered in the French hospital discharge database (2009-2015)

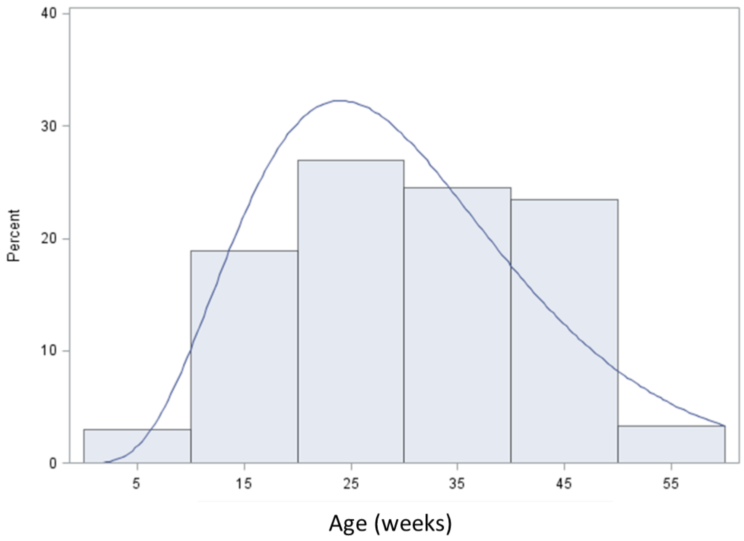

Figure S3: Age distribution of hospitalizations for intussusception fitted from the hospital stays coded K56.1 and registered in the French hospital discharge database (2009-2015)

Supplement Tables

Table S1: Model input parameters for benefit (annual number of prevented hospitalizations for RVGE before 5 years of age)

Input parameter	Reference	Mean (SD) or [95% confidence interval]	Probability distribution: family and parameters
Annual incidence of RVGE hospitalizations per 1,000 infants	a	3.1 (0.4)	Gamma shape=61.2; scale=5.09e-5
Number of RVGE deaths per year	(4)	[14-17]	Lognormal m=ln(16); s=0.046
Direct efficacy of Rotarix			
After first dose, during first year of life	(5,6)	[0.902 – 0.988]	Beta α=80.7; β=3.80
After first dose, during second and third years of life		[0.856 – 0.943]	Beta α=156.9; β=16.7
After second dose, during first year of life		[0.818 – 0.999]	Beta α=17.5; β=0.92
After second dose, during second and third years of life		[0.656 – 0.991]	Beta α=10.7; β=1.46
Direct efficacy of RotaTeq			
After first dose, during first year of life	(7)	[0.517 – 0.650]	Beta α=122.4; β=87.1
After first dose, during second and third years of life	b	[0.417 – 0.550]	Beta α=104.2; β=111.3
After second dose, during first year of life	b	[0.700 – 0.825]	Beta α=133.9; β=41.0
After second dose, during second and third years of life	b	[0.600 – 0.725]	Beta α=144.7; β=73.2
After third dose, during first year of life	(7)	[0.883 – 0.999]	Beta α=30.4; β=1.05
After third dose, during second and third years of life	(7)	[0.494 – 0.987]	Beta α=6.13; β=1.38
Basic reproduction number	(8,9)	[11 – 54]	Negative Binomial n=9; p=0.235

Abbreviations: SD, standard deviation; RVGE, rotavirus gastroenteritis

a: French National Health System (*Système National des Données de Santé*): see manuscript for details

b: Deduced by extrapolation from (7). As a 10% decrease in efficacy has been observed between the first and second year in children with the full schedule, we derived the bounds of confidence intervals hypothesizing the same 10% decrease in children with the partial schedule.

Table S2: Model input parameters for risk (annual number of vaccine-induced hospitalizations for intussusception before 1 year of age)

Input parameter	Reference	Mean (SD) or [95% confidence interval]	Probability distribution: family and parameters
Annual incidence of intussusception hospitalizations per 100,000 infants	a	28.0 (1.9)	Gamma<br>shape=214.4; scale=1.31e-6
Case-fatality rate of intussusception	(10)	0.0012 (0.0007)	Beta<br>α=3; β=2584
Relative-risk of intussusception post-dose one of Rotarix			
During first week	(11)	[2.4 – 19.0]	Lognormal<br>m=ln(6.76); s=0.52
During second and third weeks		[1.3 – 8.9]	Lognormal<br>m=ln(3.45); s=0.48
Relative-risk of intussusception post-dose two of Rotarix			
During first week	(11)	[1.1 – 7.3]	Lognormal<br>m=ln(2.84); s=0.47
During second and third weeks		[1.0 – 4.6]	Lognormal<br>m=ln(2.11); s=0.39
Relative-risk of intussusception post-dose one of RoTateq			
During first week	(11)	[3.7 – 26.4]	Lognormal<br>m=ln(9.89); s=0.49
During second and third weeks		[2.8 – 14.4]	Lognormal<br>m=ln(6.32); s=0.41
Relative-risk of intussusception post-dose two of RoTateq			
During first week	(11)	[1.2 – 6.8]	Lognormal<br>m=ln(2.81); s=0.44
During second and third weeks		[0.8 – 3.9]	Lognormal<br>m=ln(1.77); s=0.39

Abbreviations: SD, standard deviation

a: French National Health System (*Système National des Données de Santé*): see manuscript for details

Table S3: Details on vaccination schedule

	Rotarix	RotaTeq
dose 1	weeks 7 to 15	weeks 7 to 12
dose 2	weeks 11 to 23	weeks 11 to 22
dose 3		weeks 15 to 32

The vaccination schedules were assumed to be those recommended by the manufacturers, and the weekly number of vaccinated infants was considered to be constant within the recommended age intervals.

Table S4a: Estimated indirect effectiveness and annual benefits and risks of rotavirus vaccine, under various scenarios of vaccine coverage (assuming 100% Rotarix vaccines) and of efficacy waning, obtained after 20,000 simulations. Benefits, risks and BR ratios are given for hospitalization (standard font) and for death (italic font).

Vaccine coverage	Waning scenario	Indirect effectiveness, mean (sd)	Benefit<br>Number of prevented rotavirus gastroenterites			Risk<br>Number of induced intussusceptions		Benefit-Risk ratio		
			Median	2.5th-97.5th percentiles		Median	2.5th -97.5th percentiles	Median	2.5th -97.5th percentiles	
	linear	6.5% (0.4%)	1,693<br>*2.3*	1,263<br>*2.0*	2,188<br>*2.6*			292.1<br>*397.5*	156.6<br>*119.7*	578.3<br>*1,877*
10%	accelerated	5.6% (0.3%)	1,553<br>*2.1*	1,175<br>*1.9*	2,020<br>*2.4*	5.8<br>*0.006*	3.1 – 10.1<br>*0.001 – 0.02*	266.4<br>*363.1*	143.0<br>*111.4*	527.5<br>*1,725*
	absence	7.4% (0.5%)	1,809<br>*2.5*	1,361<br>*2.1*	2,331<br>*2.8*			313.2<br>*418.9*	166.7<br>*130.5*	613.5<br>*2,021*
	linear	32.4% (2.0%)	7,161<br>*9.9*	5,427<br>*8.7*	9,234<br>*11.0*			246.8<br>*336.8*	134.1<br>*105.0*	485.8<br>*1,613*
50%	accelerated	27.9% (1.6%)	6,702<br>*9.2*	5,089<br>*8.1*	8,650<br>*10.3*	29.0<br>*0.03*	15.4 – 50.0<br>*0.007 – 0.09*	231.8<br>*313.8*	124.2<br>*97.9*	453.4<br>*1,503*
	absence	36.9% (2.4%)	7,529<br>*10.4*	5,691<br>*9.1*	9,683<br>*11.5*			259.2<br>*353.1*	140.1<br>*108.1*	505.3<br>*1,686*
	linear	58.3% (3.7%)	10,530<br>*14.5*	8,049<br>*13.0*	13,490<br>*16.0*			202.0<br>*275.8*	108.4<br>*84.2*	394.2<br>*1,341*
90%	accelerated	50.2% (3.0%)	10,150<br>*14.0*	7,725<br>*12.5*	13,020<br>*15.4*	52.2<br>*0.05*	27.9– 90.5<br>*0.01 – 0.2*	193.5<br>*261.6*	104.0<br>*81.6*	379.0<br>*1,294*
	absence	66.4% (4.3%)	10,840<br>*14.9*	8,242<br>*13.4*	13,870<br>*16.4*			208.2<br>*281.2*	112.9<br>*87.9*	407.2<br>1,363

Table S4b: Estimated indirect effectiveness and annual benefits and risks of rotavirus vaccine, under various scenarios of vaccine coverage (assuming 100% Rotateq vaccines) and of efficacy waning, obtained after 20,000 simulations. Benefits, risks and BR ratios are given for hospitalization (standard font) and for death (italic font).

Vaccine coverage	Waning scenario	Indirect effectiveness, mean (sd)	Benefit<br>Number of prevented rotavirus gastroenterites			Risk<br>Number of induced intussusceptions		Benefit-Risk ratio		
			Median	2.5th-97.5th percentiles		Median	2.5th -97.5th percentiles	Median	2.5th -97.5th percentiles	
10%	linear	6.4% (0.4%)	1,660<br>*2.3*	1,253<br>*2.0*	2,141<br>*2.6*	6.5<br>*0.007*	3.8 – 10.6<br>*0.001 – 0.02*	256.5<br>*347.5*	144.3<br>*108.9*	466.7<br>*1,658*
	accelerated	5.5% (0.3%)	1,529<br>*2.1*	1,150<br>*1.8*	1,968<br>*2.4*			235.5<br>*317.5*	133.3<br>*99.0*	423.2<br>*1,492*
	absence	7.3% (0.5%)	1,769<br>*2.4*	1,332<br>*2.1*	2,293<br>*2.8*			273.6<br>*370.1*	154.4<br>*115.3*	498.6<br>*1,712*
50%	linear	32.2% (2.0%)	7,058<br>*9.7*	5,357<br>*8.5*	9,083<br>*10.9*	32.3<br>*0.03*	18.9 – 53.5<br>*0.007 – 0.10*	216.6<br>*289.5*	124.1<br>*92.4*	393.3<br>*1,357*
	accelerated	27.7% (1.6%)	6,587<br>*9.1*	4,994<br>*8.0*	8,467<br>*10.2*			204.0<br>*276.4*	116.5<br>*88.4*	368.5<br>*1,273*
	absence	36.7% (2.4%)	7,408<br>*10.2*	5,599<br>*9.0*	9,525<br>*11.4*			228.5<br>*308.1*	129.8<br>*98.3*	416.1<br>*1,435*
90%	linear	57.9% (3.7%)	10,390<br>*14.3*	7,923<br>*12.9*	13,350<br>*15.8*	58.3<br>*0.06*	33.9 – 95.4<br>*0.01 – 0.18*	178.4<br>*242.0*	101.6<br>*77.6*	321.6<br>*1,115*
	accelerated	49.9% (3.0%)	10,010<br>*13.8*	7,629<br>*12.3*	12,830<br>*15.2*			172.2<br>*229.5*	96.7<br>*73.0*	311.9<br>*1,094*
	absence	65.9% (4.3%)	10,730<br>*14.7*	8,187<br>*13.2*	13,720<br>*16.2*			183.3<br>*248.2*	104.0<br>*78.4*	332.4<br>*1,129*

Table S5a: Estimated annual benefits and benefit-risk ratios of rotavirus vaccine, under various scenarios of vaccine coverage (assuming 100% Rotarix vaccines) and of efficacy waning, in event of no indirect protection, obtained after 20,000 simulations. Benefits, risks and BR ratios are given for hospitalization (standard font) and for death (italic font).

Vaccine coverage	Waning scenario	Number of prevented rotavirus gastroenterites		Benefit-Risk ratio [a]	
		Median	2.5th-97.5th percentiles	Median	2.5th -97.5th percentiles
10%	linear	1,008<br>*1.4*	763.1 – 1,300<br>*1.2 – 1.6*	174.2<br>*239.9*	94.1 – 344.2<br>*73.8 – 1167*
	accelerated	964.2<br>*1.3*	726.0 – 1,247<br>*1.1 – 1.5*	166.0<br>*224.0*	90.1 – 322.6<br>*70.0 – 1102*
	absence	1,029<br>*1.4*	773.2 – 1,330<br>1.2 –1.6	176.9<br>*240.8*	95.7 – 344.8<br>*74.5 – 1167*
50%	linear	5,046<br>*7.0*	3,803 – 6,511<br>*6.0 – 7.8*	*a*	*a*
	accelerated	4,823<br>*6.7*	3,624 – 6,211<br>*5.7 – 7.5*		
	absence	5,148<br>*7.1*	3,872 – 6,670<br>*6.1 – 8.0*		
90%	linear	9,065<br>*12.5*	6,799 – 11,700<br>*10.8 – 14.0*	*a*	*a*
	accelerated	8,676<br>*12.0*	6,563 – 11,270<br>*10.4 – 13.4*		
	absence	9,261<br>*12.8*	7,001 – 12,040<br>*11.0 – 14.3*		

[a] Benefit-risk ratio does not depend on vaccine coverage in event of no indirect protection.

Table S5b: Estimated annual benefits and benefit-risk ratios of rotavirus vaccine, under various scenarios of vaccine coverage (assuming 100% Rotateq vaccines) and of efficacy waning, in event of no indirect protection, obtained after 20,000 simulations. Benefits, risks and BR ratios are given for hospitalization (standard font) and for death (italic font).

Vaccine coverage	Waning scenario	Number of prevented rotavirus gastroenterites		Benefit-Risk ratio [a]	
		Median	2.5th-97.5th percentiles	Median	2.5th -97.5th percentiles
10%	linear	973.9<br>*1.3*	730.6 – 1,265<br>*1.4 – 1.5*	150.4<br>*203.8*	84.8 – 273.6<br>*65.7 – 951.9*
	accelerated	934.0<br>*1.3*	698.6 – 1,216<br>*1.1 – 1.5*	143.9<br>*196.9*	*81.7* – 263.3<br>*62.3 – 924.1*
	absence	994.5<br>*1.4*	739.4 – 1,296<br>1.2 –1.5	153.6<br>*206.5*	86.2 – 280.4<br>*65.3 – 975.6*
50%	linear	4,874<br>*6.7*	3,650 – 6,317<br>*5.7 – 7.6*	*a*	*a*
	accelerated	4,666<br>*6.5*	3,506 – 6,042<br>*5.5 – 7.3*		
	absence	4,965<br>*6.9*	3,708 – 6,462<br>*5.8 – 7.7*		
90%	linear	8,773<br>*12.1*	6,543 – 11,380<br>*10.2 – 13.7*	*a*	*a*
	accelerated	8,403<br>*11.6*	6,295 – 10,880<br>*9.9 – 13.1*		
	absence	8,946<br>*12.4*	6,634 – 11,600<br>*10.4 – 14.0*		

[a] Benefit-risk ratio does not depend on vaccine coverage in event of no indirect protection.

Supplement References

1. Pivette M. Surveillance des maladies infectieuses à partir des ventes de médicaments en pharmacies. Université Paris Descartes; 2015.
2. Halloran Longini, Jr., Ira M., Struchiner, Claudio J. ME. Design and Analysis of Vaccine Studies. Statistics for Biology and Health. Springer; 2010.
3. Sabbe M, Berger N, Blommaert A, Ogunjimi B, Grammens T, Callens M, et al. Sustained low rotavirus activity and hospitalisation rates in the post-vaccination era in Belgium, 2007 to 2014. Euro Surveill. 2016 Jul 7;21(27).
4. Estimated rotavirus deaths for children under 5 years of age: 2013 [Internet]. [cited 2019 Mar 12]. Available from: https://www.who.int/immunization/monitoring_surveillance/burden/estimates/rotavirus/en/
5. Clark A, Jit M, Andrews N, Atchison C, Edmunds WJ, Sanderson C. Evaluating the potential risks and benefits of infant rotavirus vaccination in England. Vaccine. 2014 Jun;32(29):3604–10.
6. Vesikari T, Karvonen A, Prymula R, Schuster V, Tejedor JC, Cohen R, et al. Efficacy of human rotavirus vaccine against rotavirus gastroenteritis during the first 2 years of life in European infants: randomised, double-blind controlled study. Lancet. 2007 Nov;370(9601):1757–63.
7. Vesikari T, Matson DO, Dennehy P, Van Damme P, Santosham M, Rodriguez Z, et al. Safety and efficacy of a pentavalent human-bovine (WC3) reassortant rotavirus vaccine. N Engl J Med. 2006 Jan;354(1):23–33.
8. Pitzer VE, Atkins KE, de Blasio BF, Van Effelterre T, Atchison CJ, Harris JP, et al. Direct and indirect effects of rotavirus vaccination: comparing predictions from transmission dynamic models. PLoS One. 2012;7(8):e42320.
9. Stocks T, Britton T, Höhle M. Model selection and parameter estimation for dynamic epidemic models via iterated filtering: application to rotavirus in Germany. Biostatistics. 2018 Sep 27;
10. Jiang J, Jiang B, Parashar U, Nguyen T, Bines J, Patel MM. Childhood intussusception: a literature review. PLoS One. 2013;8(7):e68482.
11. Carlin JB, Macartney KK, Lee KJ, Quinn HE, Buttery J, Lopert R, et al. Intussusception risk and disease prevention associated with rotavirus vaccines in Australia's National Immunization Program. Clin Infect Dis. 2013 Nov;57(10):1427–34.
